# Characteristics of 24-hour movement behaviours and their associations with mental health in children and adolescents

**DOI:** 10.1186/s44167-023-00021-9

**Published:** 2023-06-02

**Authors:** Stuart J. Fairclough, Lauren Clifford, Denver Brown, Richard Tyler

**Affiliations:** 1 Movement Behaviours, Health, Wellbeing, and Nutrition Research Group, Department of Sport and Physical Activity, Edge Hill University, St Helens Road, Ormskirk, Lancashire, L39 4QP UK; 2 Department of Psychology, The University of Texas at San Antonio, San Antonio, TX 78249 USA

**Keywords:** Time-use, Sleep quality, Circadian, Rest-activity rhythm, Directly measured acceleration, Youth, Compositional analysis, Optimal, Goldilocks Day

## Abstract

**Background:**

Time-use estimates are typically used to describe 24-hour movement behaviours. However, these behaviours can additionally be characterised by other easily measured metrics. These include sleep quality (e.g., sleep efficiency), 24-hour rest-activity rhythmicity (e.g., between-day rhythm variability), and directly measured acceleration metrics (e.g., intensity gradient). Associations between these characteristics and youth mental health are unclear. This study aimed to [1] compare 24-hour movement behaviour characteristics by sex and age groups, [2] determine which movement behaviour characteristics were most strongly associated with mental health outcomes, and [3] investigate the optimal time-use behaviour compositions for different mental health outcomes.

**Methods:**

Three-hundred-and-one children and adolescents (age 9–13 y; 60% girls) wore accelerometers for 24-hours/day over 7-days. Overall mental health, externalising, and internalising problems were self-reported using the Strengths and Difficulties Questionnaire. 24-hour movement behaviour characteristics were categorised as time-use estimates, sleep quality, 24-hour activity rhythmicity, and directly measured acceleration. Linear mixed models and compositional data analysis were used to analyse the data in alignment with the study aims.

**Results:**

Time-use estimates, directly measured accelerations, and 24-hour rest-activity rhythm metrics indicated that children were significantly more physically active (p = .01-<0.001) than adolescents. Children were also less sedentary (p < .01), slept longer (p = .02-0.01), and had lower sleep efficiency. Boys were significantly more active than girls (p < .001) who in turn accrued more time in sleep (p = .02). The timing of peak activity was significantly later among adolescents (p = .047). Overall mental health and externalising problems were significantly associated with sleep, sedentary time, sleep efficiency, amplitude, and inter-daily stability (p = .04-0.01). The optimal time-use compositions were specific to overall mental health and externalising problems and were characterised by more sleep, light and vigorous physical activity, and less sedentary time and moderate physical activity than the sample’s mean time-use composition.

**Conclusions:**

Extracting and examining multiple movement behaviour characteristics from 24-hour accelerometer data can provide a more rounded picture of the interplay between different elements of movement behaviours and their relationships with mental health than single characteristics alone, such as time-use estimates. Applying multiple movement behaviour characteristics to the translation of research findings may enhance the impact of the data for research users.

**Supplementary Information:**

The online version contains supplementary material available at 10.1186/s44167-023-00021-9.

## Background

The paradigm of 24-hour movement behaviours recognises the inter-related influences that sleep, sedentary time (ST), and physical activity have on health and wellbeing. This approach is now well established and is reflected in the growth of national guidelines for children and adolescents (youth) that focus on 24-hour movement behaviours [1–4]. Further, advances in accelerometry-based measurement methods have corresponded with the increased number of studies reporting sleep, sedentary behaviours, and physical activity in combination [5–7]. Among youth, empirical and review studies of 24-hour movement behaviours show that achieving at least one of the movement behaviour recommendations is favourably associated with better weight status, dietary patterns, and physical, cardiometabolic, and mental health, with more favourable health outcomes associated with meeting more than one movement behaviour recommendation [8–11]. Despite these positive relationships, the most contemporary international prevalence review concluded that only 7% of youth achieve all three 24-hour movement guidelines [5].

Recently, recommendations for analytical approaches to assess associations with accelerometer-determined 24-hour movement behaviours have been made [12]. One such approach is compositional data analysis, which allows co-dependent time-use estimates of 24-hour movement behaviours to be analysed as movement behaviour compositions in relation to health outcomes [13]. In youth, cross-sectional and longitudinal associations have been reported between 24-hour movement behaviour compositional time-use and adiposity [14], fitness [14], skeletal health [15], cognitive function [16], mental health [16], and psychosocial outcomes [17]. Moreover, it was recently shown that the optimal time-use compositions for favourable physical health and mental health outcomes had relatively higher moderate-to-vigorous physical activity (MVPA) and sleep (and relatively less ST), respectively [18]. Conversely, ST and screen time were generally most strongly associated with unfavourable outcomes (e.g., internalising mental health problems, such as depression), but also positively related to cognitive health [18]. Thus, 24-hour movement behaviour compositions differentially relate to health, depending on the outcomes of interest.

In addition to time-use estimates, 24-hour movement behaviours can be characterised by other accelerometry-derived metrics. These include indicators of sleep quality [19], elements of circadian or 24-hour rest-activity rhythms [20], and directly measured movement behaviour raw acceleration metrics [21]. Sleep onset, efficiency, and regularity are example indicators of sleep quality which may be related to depression [22] and externalising mental health manifestations, such as attention-deficit problems among youth [23]. 24-hour rest-activity rhythms can be summarised by a cyclical diurnal pattern consisting of periods of resting (usually sleep), increased morning activity, relatively higher waking activity during the day, and decreased activity as the next nocturnal sleep period approaches [20]. Various parametric and non-parametric approaches have been derived from accelerometer data to characterise 24-hour rhythmicity (e.g., regularity of rest-activity cycles through consistent sleep and wake-up times) [20]. However, 24-hour rest-activity rhythms have seldom been studied in relation to youth mental health. One recent representative Dutch study found that higher intra-daily variability (i.e., increased rest-activity rhythm fragmentation, for example, nocturnal waking and day time sleep) was positively associated with internalising and externalising mental health problems in 10–11 year olds but not 13–14 year old peers [24]. This line of investigation may offer important insights to understanding relationships between 24-hour movement behaviours and mental health in youth, particularly as 24-hour activity rhythms undergo significant change in early adolescence, when a circadian phase delay is commonly observed [25].

Directly measured raw acceleration metrics can provide a picture of individuals’ 24-hour movement behaviour profiles beyond time estimated in sleep, sedentary behaviours, and physical activity [12]. This is important because how people accumulate their movement behaviours (i.e., their behaviour patterning) can influence health outcomes differently [26]. Thus, data-driven accelerometer metrics that use all available data (unlike for example, cut-point approaches that condense the activity intensity continuum into a limited number of broad categories to produce time-use estimates) can enhance our understanding of movement behaviour patterns and how they relate to different health outcomes. Expressing summary acceleration values as the daily volume and intensity distribution of movement behaviours can be studied using two directly measured raw acceleration metrics: the average acceleration (indicative of volume) and the intensity gradient (indicative of intensity distribution) [27]. These metrics have utility in describing movement behaviour volume and intensity profiles and their respective associations with health outcomes [21]. For example, health-related quality of life has been shown to be independently associated with average acceleration in youth, whereas intensity gradient was independently associated with adiposity and fitness [28].

Little is known about which 24-hour movement behaviour characteristics most strongly associate with youth mental health when the characteristics are considered collectively. Moreover, studying such characteristics together using contemporary accelerometer methods may further enhance our understanding of the interplay between the different characteristics and mental health outcomes. The aims of this study were to [1] compare 24-hour movement behaviour characteristics by sex and age groups in a sample of children and adolescents living in England, [2] determine which movement behaviour characteristics were most strongly associated with mental health outcomes, and [3] investigate the optimal time-use behaviour compositions for different mental health outcomes. The focus on mental health outcomes and optimal time-use compositions aligns the study to the Framework for Viable Integrative Research in Time-Use Epidemiology [29].

## Methods

### Participants

Participants were recruited from primary and secondary schools across two studies in the West Lancashire region of northwest England. The first study examined associations between 24-hour movement behaviours, mental health, and cognitive function. It took place in autumn 2019-20 and involved 382 participants aged 9–10 and 12–13 years. The second study was a school-based intervention feasibility trial, which occurred in autumn 2022 involving 108 participants aged 9–10 years. Participants in both studies provided written informed parental consent following approval from the Edge Hill University Research Ethics Committee (#SPA-REC-2018-007 and #ETH2122-0062) and data were pooled for the current analyses.

### Anthropometric and demographic measures

Body mass and height were measured to the nearest 0.1 kg and 0.1 cm, respectively following standard procedures [30]. Body mass index (BMI) and BMI z-scores (BMIz) were calculated for each participant [31] and international age- and sex-specific BMI cut-points applied to determine weight status [32]. For all measurements, participants wore light clothing with shoes removed. Participants’ dates of birth, home post codes, and ethnicity were obtained from the schools’ information management systems. Decimal age and 2019 English Indices of Multiple Deprivation (EIMD) scores [33] were calculated using data collection dates and home post codes, respectively. EIMD scores provide an area-level relative measure of deprivation based on income, employment, education, health, crime, housing, and living environment. Area-level socioeconomic status (SES) was represented by the EIMD decile score for each participant.

### Mental health

Mental health was measured in classrooms using the Strengths and Difficulties Questionnaire (SDQ) [34]. All participants completed the SDQ, which includes five subscales related to perceived emotional problems, behavioural problems, hyperactivity, peer relationship problems, and prosocial behaviour in the last six months. Each subscale consists of five items which are scored on a 3-point scale ranging from 0 (‘not true’) to 2 (‘certainly true’). Scores for each subscale are computed by summing their respective items and range from 0 to 10. A total difficulties score reflective of overall mental health can also be computed by summing the four mental health problems subscales, ranging from 0 to 40, with higher scores reflecting increased mental health problems. Prosocial behaviour is not included within the total difficulties score as it is the positive mental health subscale on the SDQ. When the SDQ is used with community samples it is recommended that the broad constructs of internalising problems (emotional problems and peer relationships), externalising problems (behavioural problems and hyperactivity), and overall mental health are reported [35]. Computed scores for each of the five subscales and the total difficulties scale can be classified on a four-band categorization: close to average, slightly raised, high, and very high [36]. Support for the scale’s construct validity has been demonstrated through correlations between scores and clinical diagnoses of relevant mental health disorders [34]. Internal consistency of the SDQ for internalising problems, externalising problems, and overall mental health was Cronbach’s α = 0.56, 0.68, and 0.75, respectively.

### Assessment of 24-hour movement behaviours

Participants were asked to wear ActiGraph GT9X triaxial accelerometers on their non-dominant wrist for 24-hours per day over 7 consecutive days. The devices were initialised to record at 100 Hz and the subsequent data were downloaded using ActiLife (versions 6.13.4). The raw data files (gt3x format) were processed in R using package GGIR version 2.8-2 [37]. Signal processing included autocalibration using local gravity as a reference [38], detection of implausible values, and identification of non-wear. Non-wear was imputed by default in GGIR whereby invalid data were imputed by the average at similar time points on other days of the week [39]. The raw triaxial accelerometer signals were converted to one summary measure of acceleration (Euclidean Norm Minus-One; ENMO) expressed in milligravitational units (m*g*) [39]. ENMO values were reduced to 5-s epochs and averaged over each of the 7 monitored days to represent average acceleration. Participants’ accelerometer data were included in the analytical sample if valid wear was recorded for 24-h·d^–1^ (i.e., GGIR output variable *complete_24hcycle* = 1.0) on at least three weekdays and one weekend day, and if post-calibration error was < 10 m*g*. All 24-hour movement behaviour characteristic outcomes were calculated in GGIR as the average weighted week (i.e., 5:2 ratio).

### 24-hour movement behaviour characteristics

Time-use estimates. Youth-specific non-dominant wrist ENMO cut-points of 48 m*g* [40], 201 m*g*, and 707 m*g* [41] defined the estimated upper threshold of ST and lower threshold of light physical activity (LPA), moderate physical activity (MPA), and vigorous physical activity (VPA), respectively. Sleep duration was estimated using a polysomnography-validated accelerometer algorithm based on the distribution of change in the z-angle (i.e., corresponding to the axis positioned perpendicular to the skin surface) [19].

Sleep quality. A range of GGIR-derived metrics were used to represent sleep quality. These were sleep efficiency (percentage of time in bed spent asleep), time of sleep onset, and mean number of awakenings per night.

**Fig. 1:**
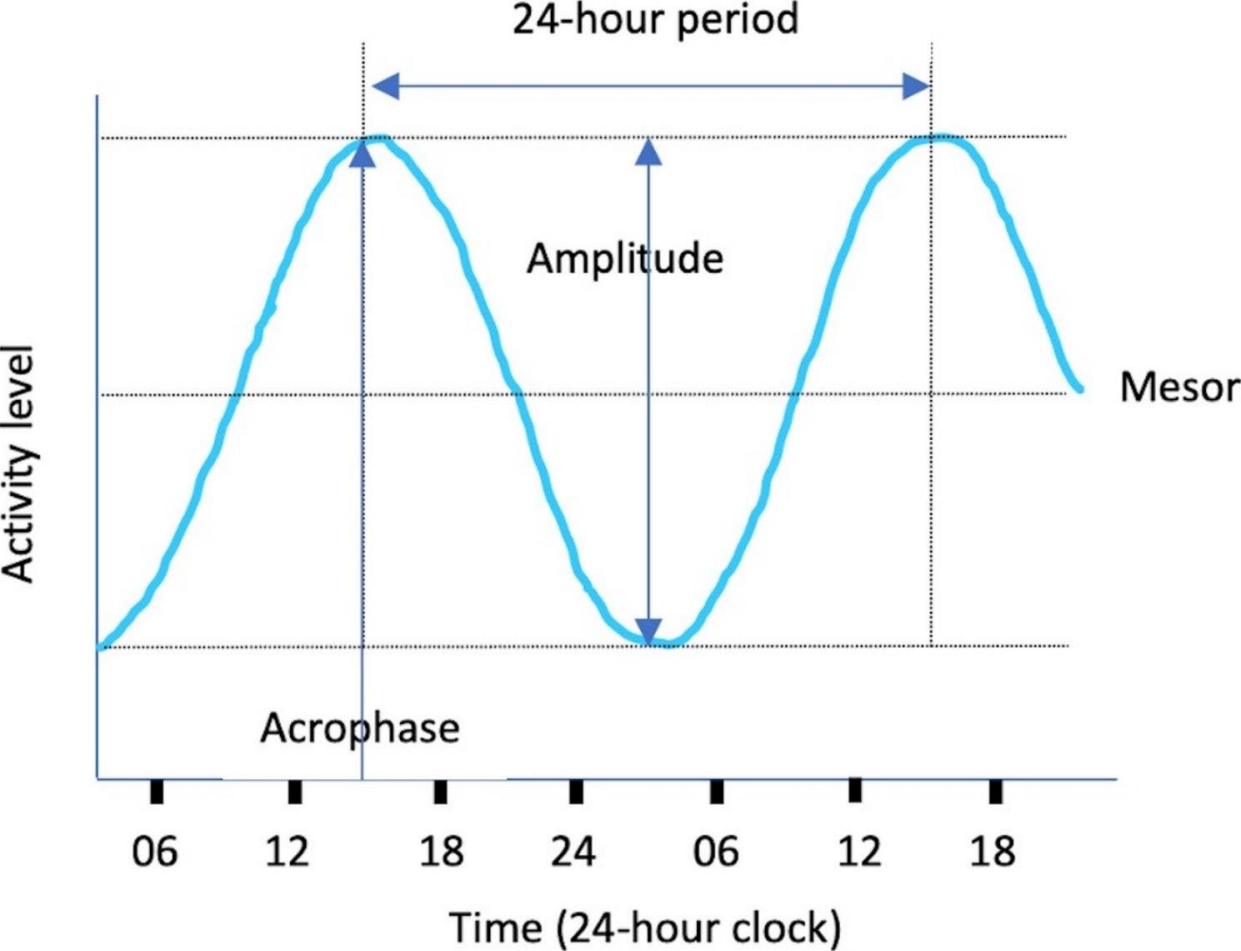
Description of the extended cosinor model and associated 24-hour rest-activity rhythm metrics

24-hour rest-activity rhythms. We used a combination of parametric and non-parametric metrics to describe 24-hour rest-activity rhythms. Extended cosine parametric methods are inverse-logit transformations of the standard cosine curve which tend to provide a better model fit than the simple cosinor algorithm [20]. Extended cosinor model metrics were calculated in GGIR through the dependent package ActCr [42] and expressed as log m*g* + 1. Figure 1 provides a visual example of the cosinor model and Table 1 presents descriptions of the 24-hour rest-activity rhythm metrics used in this study. The extended cosine metrics of interest were mesor (mean activity level), amplitude (difference between the minimum and maximum activity), and acrophase (timing of the rhythm peak) [43]. Non-parametric metrics were inter-daily stability (IS; consistency of the 24-hour rest-activity rhythm between days; range 0–1), intra-daily variability (IV; within-day fragmentation of the 24-hour rest-activity rhythm; range 0–2), mean activity level (in m*g*) during the most active 10-hours of the 24-hour period (M10), and mean activity level (in m*g*) during the least active 5-hours of the 24-hour period (L5).

**Table 1:** Descriptions of the 24-hour rest-activity rhythm metrics used in this study

Metric	Description	Explanation
Mesor (Midline estimating statistic of rhythm)	Mean level of activity over the 24-hour period	A person with a higher mesor has recorded more average activity across the 24-hour period than someone with a lower mesor
Amplitude	Difference between the peak activity level and minimum activity level	A person with a higher amplitude has recorded more overall maximum activity relative to their minimum activity and has higher overall rhythmicity (more rhythmic changes) than someone with a lower amplitude
Acrophase	Timing of peak activity in the 24-hour period	A person’s peak activity occurs later in the 24-hour period when acrophase is at a later time than someone with an earlier acrophase. Later acrophase may reflect a more delayed activity phase in the 24-hour period.
Inter-daily stability (IS)	Day-to-day variability of the 24-hour rest-activity rhythm	A person with consistent daily rest-activity rhythms over consecutive days has a higher IS than someone with a weaker adherence to the circadian rhythm between days.
Intra-daily variability (IV)	Within-day fragmentation of the 24-hour rest-activity rhythm and transitions between rest and activity	A person with a lower IV has a relatively stronger rest-activity rhythm (e.g., better sleep efficiency, lower fragmentation of rest-activity rhythms) than someone with higher IV.
M10	Mean activity level during the most active 10-hours of the 24-hour period	A person with a higher M10 would average more activity during waking hours than someone with a lower M10 value.
L5	Mean activity level during the least active 5-hours of the 24-hour period	A person with a lower L5 would average less activity during resting (usually nocturnal) hours than someone with a higher L5 value. A higher L5 is reflective of less restful sleep.

Directly measured raw acceleration metrics. Activity volume was expressed as the mean average acceleration over 24-hours, and activity intensity distribution was represented by the intensity gradient metric [21]. Intensity gradient is based on the relationship between log values for intensity (i.e., incremental intensity bins, 0–25 m*g*, 25–50 m*g*, etc.) and time (i.e., accumulated time in each intensity bin), and is always negative, reflecting less time being accumulated in increasing intensity bins [21].

### Data analysis

For aim 1, sex and age-group comparisons of 24-hour movement behaviour characteristics were assessed using linear mixed models. Age groups were defined as children (age 9–10 years) and adolescents (age 12–13 years). All models accounted for school-level clustering and were adjusted for SES. Analyses of time-use estimates and directly measured raw acceleration metrics were additionally adjusted for BMIz.

For aim 2, associations between mental health outcomes and the five movement behaviour time-use estimates were analysed using ‘one-for-remaining’ compositional data analysis using the R package compositions (v. 1.40-5) [44]. Five-part time-use compositions were expressed as five specific sets of four isometric log-ratios (ILRs) [45], which were used in multivariate linear regression models. Overall mental health, internalising problems, and externalising problems were the dependent variables, and the composition ILRs were the explanatory variables. Each set of ILRs contained one ILR1 (i.e., the first pivot coordinate), which captured the time in one time-use movement behaviour relative to all the remaining behaviours (i.e., the geometric mean of the remaining behaviours), ensuring that each of the five behaviours were considered against all the remaining ones. The models accounted for school-level clustering and were adjusted for sex, age, SES, and BMIz, which are established influences on movement behaviours [16, 46]. If the ANOVA table of the model fit showed that the set of movement behaviour ILRs was significantly associated with the mental health outcomes, follow-up analyses were performed. These examined the relationship of each movement behaviour (relative to all other movement behaviours) with the mental health outcomes [13]. Mental health associations with the other 24-hour movement behaviour characteristics (sleep quality, 24-hour rest-activity rhythms, and directly measured raw acceleration metrics) were determined using linear mixed models. All association analyses accounted for school-level clustering and were adjusted for sex, SES, and age (directly measured raw acceleration metrics were additionally adjusted for BMIz).

The analysis for aim 3 used the methods described by Dumuid and colleagues [18] to determine optimal time-use compositions for the mental health outcomes that were significantly associated with the time-use compositions generated for aim 2. Briefly, predictive compositions were generated to represent every possible 10-minute combination of movement behaviours recorded by the participants. The time-use limits of these predicted compositions were truncated at ± 3 SD. The resultant time-use ranges used for the predicted compositions (min·day^− 1^) were sleep = 306–631, ST = 172–900, LPA = 132–647, MPA = 9-134, and VPA = 0–33. Linear regression models adjusted for sex, age, SES, and BMIz were used to estimate the mental health outcome for each predictive composition. The optimal time-use composition for each mental health outcome was the compositional mean of the ‘optimal time-use zone’, which was defined as the compositions associated with the most favourable 5% of the outcome (i.e., at or above the 95% percentile) [18]. Analyses for aims 1–3 were performed with the compositions (v. 1.40-5) [44], lmerTest (v. 3.1-3) [47], and car (v. 3.0–12) [48] R packages, and model diagnostics were undertaken using the performance package (version 0.9.0) [49].

## Results

Written informed consent was provided for 490 participants, with 23 absent from school on the day of data collection. Of the remaining 467 participants who wore the accelerometers, 120 did not average 24-h⋅d^− 1^ of valid wear. From the remaining 347 participants, 46 did not have 24-h⋅d^− 1^ valid wear for at least 3 weekdays and 1 weekday and were removed, leaving an analytical sample of n = 301. No significant differences were observed between the included and excluded participants for BMIz (p = .87), project (p = .14), and sex (p = .51). Excluded participants were older than included peers (11.7±1.7 y vs. 10.7±1.3 y, p < .001), and were more likely to be from lower SES households (i.e., EIMD deciles 1–3 = 29.20% vs. 11.4%; EIMD deciles 4–7 = 33.3% vs. 30.2%; p < .001).

Participants’ descriptive characteristics are presented in Table 2. Both the child and adolescent groups included a lower proportion of boys (40.5% and 38.8%) than girls (59.5% and 61.2%). Relatively more children (30.4%) than adolescents (23.5%) were classified as overweight/obese, and 93.5% of all participants were of White British ethnicity. Scores on the Strengths and Difficulties Questionnaire were higher in children compared to adolescents for overall mental health, externalising problems, and internalising problems. The mean values for overall mental health were in the ‘close to average’ categorisation [50], suggesting low risk of clinically relevant mental health problems in the sample.

**Table 2:** Descriptive characteristics of the sample (Mean (SD) or percentage)

	All participants			Children			Adolescents		
	Boys n = 120	Girls n = 181	All n = 301	Boys n = 75	Girls n = 110	All n = 185	Boys n = 45	Girls n = 71	All n = 116
Age (years)	11.0 (1.6)	11.1 (1.7)	11.1 (1.6)	9.9 (0.4)	9.8 (0.4)	9.8 (0.4)	13.0 (0.3)	13.1 (0.3)	13.1 (0.3)
Height (cm)	147.4 (11.8)	146.0 (12.8)	146.5 (12.4)	140.4 (7.3)	138.5 (8.1)	139.3 (7.8)	159.0 (8.2)	157.4 (9.8)	146.5 (12.4)
Weight (kg)	41.5 (12.1)	42.1 (13.3)	41.8 (12.8)	36.2 (9.2)	35.9 (9.6)	36.0 (9.4)	50.4 (11.1)	51.6 (12.6)	51.1 (12.0)
BMI (kg⋅m^2^)	18.7 (3.4)	19.4 (3.9)	19.1 (3.7)	18.1 (3.2)	18.5 (3.4)	18.3 (3.4)	19.8 (3.4)	20.7 (4.2)	20.4 (3.9)
BMIz	0.62 (1.24)	0.46 (1.21)	0.48 (1.22)	0.54 (1.32)	0.48 (1.21)	0.50 (1.25)	0.48 (1.09)	0.44 (1.22)	0.46 (1.17)
Weight status									
Healthy weight (%)	74.2	71.1	72.3	73.3	67.0	69.6	75.6	77.1	76.5
Overweight/obese (%)	25.8	28.9	27.7	26.7	33.0	30.4	24.4	22.9	23.5
EIMD decile	6.6 (2.8)	6.7 (3.0)	6.7 (2.9)	6.6 (3.0)	6.7 (3.2)	6.6 (3.1)	6.5 (2.5)	6.8 (2.7)	6.7 (2.6)
Ethnicity									
White British (%)	94.2	94.5	94.4	96.0	91.7	93.5	91.1	98.6	95.7
Other (%)	5.8	5.4	5.6	4.0	8.3	6.5	8.9	1.4	4.3
SDQ responses									
Total difficulties	11.5 (6.2)	10.7 (5.6)	11.1 (5.8)	12.3 (6.2)	10.9 (5.6)	11.5 (5.9)	10.1 (6.1)	10.5 (5.6)	10.3 (5.8)
Externalising problems	6.2 (3.6)	5.1 (3.3)	5.6 (3.5)	6.8 (3.5)	5.3 (3.2)	5.9 (3.4)	5.4 (3.6)	5.0 (3.4)	5.1 (3.5)
Internalising problems	5.3 (3.7)	5.6 (3.4)	5.5 (3.5)	5.6 (3.7)	5.6 (3.5)	5.6 (3.6)	4.8 (3.6)	5.5 (3.3)	5.2 (3.4)

Note. BMI = body mass index; BMIz = body mass index z-score; EIMD = English Indices of Multiple Deprivation; SDQ = Strengths and Difficulties Questionnaire

### Aim 1

Adjusted means for the 24-hour movement behaviour characteristics are described in Table 3. Children engaged in significantly more sleep, MPA, and VPA than adolescents (p = .02 to p = .01), who spent more time being sedentary (p = .01). Girls slept longer than boys (p = .02) who did more MPA and VPA (p < .001). Sleep efficiency was highest among adolescents (p = .001) who had fewer awakenings and later sleep onset than children. Of the 24-hour rest-activity rhythm characteristics, children recorded higher mesor (mean activity) (p = .01) and M10 (mean activity during the most active 10 h) values than adolescents (p = .01), who had significantly later timing of peak activity (acrophase) (p = .047). Boys recorded higher amplitude (difference between peak and minimum activity) (p < .001) and M10 values than girls (p < .001). For directly measured acceleration metrics, average acceleration (p = .02) and intensity gradient were higher in children compared to adolescents (p = .01), and in boys compared to girls (p < .001).

**Table 3:** Sex and age-group comparisons of 24-hour movement behaviour characteristics (adjusted means and 95% CI)

	All participants		Children			Adolescents		
	Boys	Girls	Boys	Girls	All	Boys	Girls	All
Time-use estimates								
Sleep (min⋅d^− 1^)	450.5 (442.1, 458.8)	463.5* **(456.8, 470.3)**	465.9 (455.7, 476.2)	486.4 (477.9, 494.9)	476.2*** (469.5, 482.8)	435.0 (421.7, 448.2)	440.6 (430.1, 451.2)	437.8 (429.4, 446.3)
ST (min⋅d^− 1^)	569.2 (532.1, 606.3)	566.1 (528.3, 603.9)	512.8 (482.4, 543.2)	514.0 (485.5, 542.4)	513.4 (486.4, 540.4)	619.5 (550.0, 688.9)	624.5 (555.8, 693.2)	622.0** **(554.1, 689.9)**
LPA (min⋅d^− 1^)	283.6 (250.4, 316.7)	286.0 (253.2, 318.8)	305.8 (280.4, 331.1)	303.8 (279.6, 327.9)	304.8 (281.4, 328.2)	261.4 (200.0, 322.8)	268.2 (207.1, 329.2)	264.8 (204.2, 325.3)
MPA (min⋅d^− 1^)	60.6*** **(54.7, 66.5)**	48.9 (43.3, 54.6)	67.4 (62.0, 72.8)	55.5 (50.7, 60.4)	61.4* **(57.1, 65.8)**	53.8 (43.12, 64.4)	42.4 (32.0, 52.8)	48.1 (37.9, 58.3)
VPA (min⋅d^− 1^)	10.5*** **(9.1, 12.0)**	6.5 (5.1, 7.9)	12.9 (11.5, 14.3)	8.8 (7.6, 10.0)	10.9** **(9.8, 11.9)**	8.1 (5.6, 10.7)	4.3 (1.7, 6.8)	6.2 (3.6, 8.8)
Sleep quality								
Sleep efficiency (%)	87 (86, 87)	88 (87, 88)	86 (85, 87)	87 (86, 88)	86 (86, 87)	88 (86, 89)	88 (97, 89)	88** **(87, 89)**
Night awakenings	20.5 (19.6, 21.4)	20.0 (18.3, 21.7)	21.2 (20.3, 22.0)	21.0 (20.3, 21.8)	21.1 (20.5, 21.7)	19.9 (17.9, 21.9)	19.0 (4.7, 33.3)	19.4 (-250.0, 289.0)
Sleep onset time (decimal hours)	22.42 (22.06, 22.78)	22.37 (21.79, 22.94)	21.66 (21.35, 21.96)	21.74 (21.47, 22.01)	21.70 (21.45, 21.95)	23.2 (22.4, 24.0)	23.0 (20.5, 25.5)	23.09 (10.36, 35.82)
24-h rest-activity rhythms								
Mesor (log m*g* + 1)	2.31 (2.18, 2.44)	2.31 (2.19, 2.44)	2.50 (2.40, 2.61)	2.47 (2.37, 2.57)	2.49** **(2.39, 2.58)**	2.12 (1.89, 2.36)	2.16 (1.93, 2.39)	2.14 (1.91, 2.37)
Amplitude (log m*g* + 1)	2.78*** **(2.61, 2.95)**	2.35 (2.19, 2.52)	2.83 (2.65, 3.01)	2.38 (2.23, 2.54)	2.61 (2.48, 2.74)	2.73 (2.42, 3.03)	2.33 (2.00, 2.65)	2.53 (2.17, 2.88)
Acrophase time (decimal hours)	14.55 (14.31, 14.78)	14.58 (14.35, 14.81)	14.28 (14.09, 14.48)	14.38 (14.20, 15.46)	14.33 (14.16, 14.50)	14.81 (14.38, 15.24)	14.78 (14.35, 15.21)	14.79* **(14.37, 15.22)**
IS	0.54 (0.47, 0.60)	0.52 (0.46, 0.58)	0.52 (0.47, 0.57)	0.52 (0.47, 0.56)	0.52 (0.47, 0.56)	0.56 (0.44, 0.68)	0.52 (0.41, 0.64)	0.54 (0.42, 0.66)
IV	0.77 (0.70, 0.85)	0.81 (0.74, 0.89)	0.80 (0.74, 0.86)	0.82 (0.76, 0.87)	0.81 (0.75, 0.86)	0.75 (0.61, 0.89)	0.81 (0.68, 0.95)	0.78 (0.64, 0.92)
L5 (m*g*)	5.1 (2.4, 7.8)	5.1 (2.5, 7.8)	6.4 (4.5, 8.3)	6.4 (4.5, 8.2)	6.4 (4.5, 8.2)	3.8 (-1.3, 8.8)	3.9 (-1.1, 9.0)	3.9 (-1.2, 8.9)
M10 (m*g*)	85.0*** **(78.6, 91.5)**	71.3 (65.0, 77.6)	92.8 (86.7, 98.9)	80.6 (75.2, 86.0)	86.7* **(81.9, 91.5)**	77.3 (50.4, 65.6)	62.0 (50.4, 73.5)	69.6 (58.2, 81.0)
Directly measured acceleration								
AvAcc (m*g*)	44.9*** **(41.1, 48.6)**	39.5 (35.9, 43.1)	49.0 (45.8, 52.3)	43.9 (41.0, 46.9)	46.5* **(43.8, 49.2)**	40.7 (33.9, 47.5)	35.1 (28.4, 41.7)	37.9 (31.3, 44.5)
Intensity gradient	-2.03*** **(-2.08, -1.97)**	-2.12 (-2.17, -2.06)	-1.96 (-2.01, -1.92)	-2.04 (-2.08, -2.00)	-2.00* **(-2.04, -1.96)**	-2.09 (-2.19, -1.99)	-2.19 (-2.29, -2.09)	-2.14 (-2.24, -2.05)

Note. *p < .05, **p < .01, ***p < .001

Analyses adjusted for school-level clustering and SES. BMIz included as an additional covariate for analysis of LPA, MPA, VPA, AvAcc, and IG. ST = sedentary time; LPA = light physical activity; MPA = moderate physical activity; VPA = vigorous physical activity IS = inter-daily stability; IV = intra-daily variability; L5 = activity level during the least active 5 h; M10 = activity level during the most active 10 h, AvAcc = average acceleration

### Aim 2

Model results for all aim 2 analyses are presented in Additional file 1.

Time-use estimates. The geometric means and variation matrix for the average time-use composition are presented in Additional file 2. The average time-use composition was significantly associated with overall mental health (p = .049) and externalising problems (p = .03) but not internalising problems. ST was positively associated with overall mental health (β = 4.7, 95%CI = 1.4, 8.0, p = .01) and externalising problems (β = 2.6, 95%CI = 0.6, 4.6, p = .01), while these outcomes were negatively associated with sleep (overall mental health, β=-6.0, 95%CI=-10.9, -1.1, p = .01; externalising problems, β = 3.9, 95%CI=-6.6, -1.2, p = .01). In both models the correlations between covariates were low (VIF range = 1.06–1.56).

Sleep quality. Sleep efficiency was negatively associated with overall mental health (β=-18.0, 95%CI=-34.9, -1.1, p = .04) and externalising problems (β=-12.8, 95%CI=-22.6, -3.0, p = .01), but no other significant associations with sleep quality characteristics were observed. As sex was a significant predictor in the externalising problems model, sex-stratified analyses were performed. These revealed a significant negative association for sleep efficiency among boys (β=-17.0, 95%CI=-32.7, -1.3, p = .04) but not girls. For these models, VIF ranges were 1.01–1.13.

24-hour rest-activity rhythms. Amplitude was positively associated with overall mental health (β = 1.0, 95%CI = 0.02, 2.0, p = .03) and externalising problems (β = 0.7, 95%CI = 0.1, 1.3, p = .02). Conversely, there were negative associations between IS and overall mental health (β=-9.9, 95%CI=-18.3, -1.5, p = .02) and externalising problems (β=-6.7, 95%CI=-11.6, -1.8, p = .009). VIF ranged from 1.11 to 3.12.

Directly measured acceleration. Neither average acceleration or intensity gradient were significantly associated with overall mental health and externalising problems. The VIF values ranged from 1.05 to 1.65.

### Aim 3

**Fig. 2:**
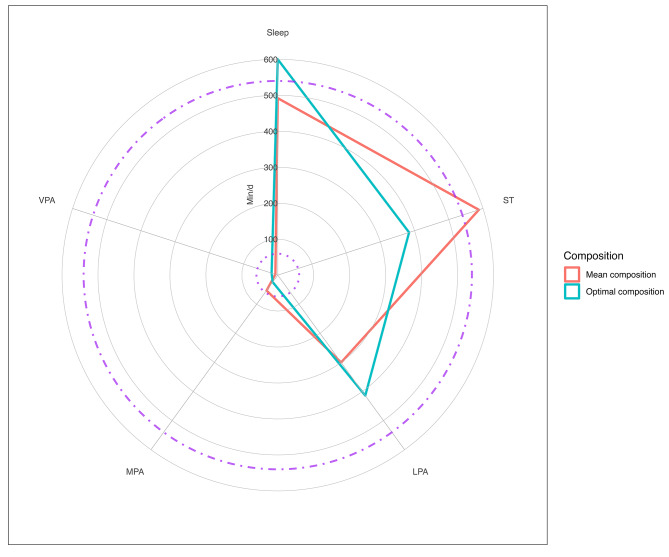
Radar plot of optimal time-use compositions for overall mental health compared to the mean sample composition. Note. The dashed lines indicate the recommended minimum sleep duration (540 min•night^− 1^) and the dotted lines indicate the recommended minimum MVPA (60 min•day^− 1^ averaged over the week). ST = sedentary time, LPA = light physical activity, MPA = moderate physical activity, VPA = vigorous physical activity, MVPA = moderate-to-vigorous physical activity

**Fig. 3:**
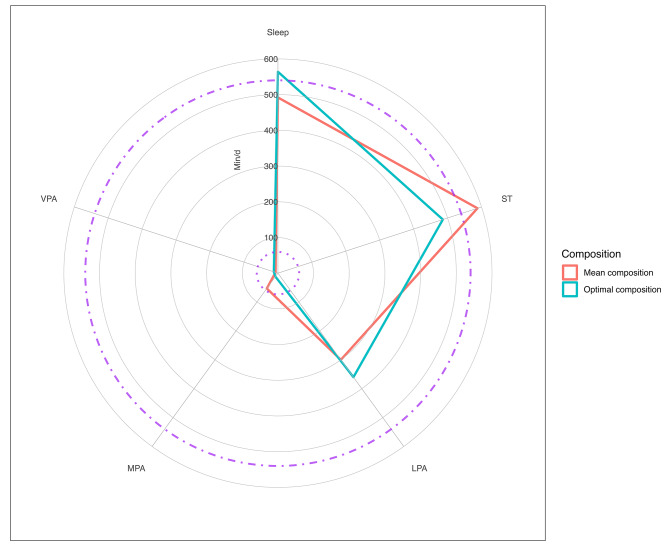
Radar plot of optimal time-use compositions for externalising problems compared to the mean sample composition. Note. The dashed lines indicate the recommended minimum sleep duration (540 min•night^− 1^) and the dotted lines indicate the recommended minimum MVPA (60 min•day^− 1^ averaged over the week). ST = sedentary time, LPA = light physical activity, MPA = moderate physical activity, VPA = vigorous physical activity, MVPA = moderate-to-vigorous physical activity

The optimal time-use compositions were different for overall mental health and for externalising problems. The radar plots [51] show that for overall mental health the optimum composition was characterised by relatively long sleep duration (10 h) and shorter ST (6.5 h) and LPA (6.9 h), with MPA and VPA totalling 43 min (Fig. 2). The best externalising problems scores were associated with relatively long sleep and ST (9.4 and 9.1 h, respectively), 6 h of LPA, and less than 30 min of MPA and VPA (Fig. 3). When compared to the sample mean time-use composition (Additional file 2) the optimal compositions included longer durations of sleep, LPA, and VPA, and less ST and MPA.

## Discussion

This study compared children’s and adolescents’ 24-hour movement behaviour characteristics, examined associations between these characteristics and mental health, and described the optimal 24-hour time-use behaviour compositions for mental health. Children were more physically active, less sedentary, slept longer, and had lower sleep efficiency than adolescents. Based on time-use estimates, 24-hour rest-activity rhythm metrics, and directly measured accelerations, boys were more active than girls who in turn accrued more time in sleep. Overall mental health and externalising problems were significantly associated with sleep duration, ST, sleep efficiency, amplitude, and IS. The optimal time-use compositions were specific to the different mental health outcomes and were characterised by more sleep, LPA, and VPA, and less ST and MPA than the sample’s mean time-use composition.

### Aim 1

Boys and children accumulated significantly more MPA and VPA, recorded greater activity volume, a higher intensity profile, and had superior M10 values than girls and adolescents, respectively, which concurs with earlier studies [28, 52, 53]. Children also had higher mesor values than adolescents which indicate higher activity across the day [20] which are consistent with the age-related differences in M10 values and average acceleration [53, 54]. This also reflects the additional 109 min•day^− 1^ of ST observed in adolescents compared to children, which offset the lower levels of sleep and physical activity in the older group. Such differences in activity have been attributed to various factors including timing and tempo of maturation [55], psychosocial reinforcement [56, 57], as well as changes to the 24-hour rest-activity rhythm [25]. The sex differences in physical activity outcomes were also reflected by the higher amplitude values for boys, which represent higher maximum activity and more robust 24-hour rest-activity rhythmicity [20].

Sleep duration was significantly longer in girls and children compared to boys and adolescents, respectively, which aligns with other empirical accelerometry studies [16, 24, 58] and a 2018 meta-analysis of accelerometer-assessed sleep [59]. A recent narrative review however, concluded that the direction of sex differences in children’s and adolescents’ sleep duration is equivocal and largely influenced by methodological differences and participant characteristics [60]. Age-related differences in sleep are more consistent [59] and reflect the delay in the 24-hour rest-activity rhythm sleep phase during adolescence (i.e., by later sleep and waking times [24, 25]). This was observed in our sample, with adolescents’ sleep onset around 1.4 h later than the children’s, while waking times on most days were similar to accommodate the school day starting around 09:00 for both age groups. Furthermore, adolescents’ acrophase timing was significantly later than children’s, indicating later timing of peak activity, and reflecting a more delayed activity phase following nocturnal sleep [20]. Adolescents had 88% sleep efficiency which was slightly, yet significantly higher than the 86% recorded for children. These differences concur with previous research [24], although both values were above the ≥ 85% sleep efficiency threshold for good sleep quality recommended by the US National Sleep Foundation so the difference observed may not have clinical relevance [61]. Conversely, sleep durations for both groups were less than the 540 and 480 min per night minimum ranges recommended for optimal health in children and adolescents, respectively [62]. These seemingly contradictory findings highlight the multidimensional nature of sleep and underscore the utility of using complementary indicators of sleep quality.

### Aim 2

In our sample the unadjusted mental health outcomes were higher in children than adolescents. This is counter to typically observed age-related differences in mental health problems among youth [63–65]. As reported by others [66, 67], it is possible that mental health issues resulting from the COVID-19 pandemic and lockdown restrictions in the study 2 children may have continued and contributed to the higher than anticipated mental health outcomes in this group. We cannot though speculate beyond this, and importantly the total difficulties SDQ scores (i.e., overall mental health) for both children and adolescents were in the ‘close to average’ range, indicating that the observed differences were not clinically relevant in either group [36].

The compositional analysis of movement behaviour time-use estimates revealed that overall mental health and externalising problems were negatively associated with sleep and positively associated with ST, relative to the remaining behaviours. These findings are broadly consistent with previous literature although the relationships between mental health and sleep or ST are quite nuanced. For example, how ST is defined influences the magnitude of associations, with relatively strong and consistent evidence observed for screen-based ST [11, 68, 69]. In our study total ST was estimated from low levels of wrist acceleration (i.e., < 48 m*g*) [40]. Such cut-point approaches are subject to misclassification of ST as stationary standing or LPA and vice-versa and provide no information about type of sedentary behaviours performed (e.g., passive versus active screen time). However, our accelerometer data were reflective of weekday and weekend activities and so it is likely that a representative range of sedentary behaviours, such as TV viewing, gaming, computer and mobile phone use, and studying were captured. Similarly, associations between sleep duration and mental health outcomes can be somewhat dependent on exposure and outcome measurement methods and amount of sleep recorded. For example, in Dutch children and adolescents, significant associations were recently observed between self-reported sleep and externalising problems, internalising problems, and dysregulation profile, but these associations were non-significant and much smaller based on wrist-accelerometer derived sleep [24]. Moreover, the beneficial influence of sleep on mental health may be strongest among youth who have insufficient sleep, with diminishing returns for those who have more sleep than is necessary for health [68]. Nonetheless, recent reviews have concluded that children and adolescents who meet sleep guidelines are more likely to have better mental health outcomes than peers who sleep less [7, 11]. Interestingly, achieving recommended levels of sedentary screen time and sleep were also reported to be more strongly associated with mental health than meeting physical activity guidelines [11]. This observation is reflected in our findings whereby LPA, MPA, VPA, average acceleration, intensity gradient, and M10 metrics were not significantly associated with any mental health outcomes, but sleep, ST, sleep quality, and 24-hour rest-activity rhythm metrics were.

Sleep efficiency was inversely associated with overall mental health and externalising problems, which is consistent with the associations observed when sleep duration was the exposure. However, the associations between sleep efficiency and externalising problems were only evident among boys. This may be partly explained by the 18% difference in boys’ and girls’ externalising problems scores (boys > girls) which mirror SDQ norms for UK youth [70]. Furthermore, sleep efficiency is influenced by number of night awakenings (in our analysis, p = .06 and p = .05 for associations with overall mental health and externalising problems, respectively) which was recently shown to be more strongly associated with externalising problems in English boys compared to girls in the nationally-representative Millennium Cohort Study [71]. These results emphasise the potential importance of sleep quality for boys reducing the risk of hyperactivity and conduct problems. Conversely, non-significant associations between accelerometer-assessed sleep efficiency and youth externalising problems were recently reported in children and adolescents from the Netherlands [24]. The study authors suggest that assessing sleep outcomes using self-report methods better captures neuronal domains of sleep that relate more to mental health problems, and which may not be captured by accelerometers [24]. On the other hand, accelerometers reduce much of the measurement error associated with self-reported behaviours and allow relatively accurate estimations of important sleep quality metrics. Clearly, there is a need for consistent and valid methodologies to be used to allow meaningful comparisons of sleep metrics between studies.

24-hour rest-activity rhythm amplitude was positively associated with overall mental health and externalising problems. The interpretation is that participants with greater maximum activity and more robust rest-activity patterns (i.e., higher activity in the day and lower activity during sleep) were likely to report higher scores for the hyperactivity and conduct items on the SDQ. This relationship seems counterintuitive because higher activity and more stable rest-activity rhythmicity are desirable for health and wellbeing [72]. Moreover, IS is indicative of between-day consistency of the 24-hour rest-activity cycle and was negatively associated with overall mental health and externalising problems. This relationship is also in the oppositive direction to what might be expected, given that poorer synchronisation of rest-activity rhythms to external zeitgebers (i.e., environmental cues for the 24-hour clock) is undesirable for mental health. For example, low IS values may reflect irregular bedtimes, which have previously been associated with overall mental health problems [73] and poorer cognitive performance among English children [74]. As the SDQ scores were not clinically relevant [36] the potential implications of these results are unclear and warrant further investigation with a sample providing more heterogenous SDQ scores.

No significant associations were observed between internalising problems and any of the 24-hour movement behaviour characteristics. We have previously reported positive associations between ST and internalising problems [16] and recent systematic reviews have shown how meeting guidelines for sleep, sedentary screen time, and physical activity are associated with reduced odds of internalising problems, like depressive symptoms, with sleep and screen time potentially more important in children compared to adolescents [7, 11]. We speculate that the relatively low SDQ scores for emotional and peer problems may have caused a ceiling effect for the internalising problems construct and therefore limited the strength of associations with some of the 24-hour movement behaviour characteristics. The lack of observed associations between time-use physical activity estimates, directly measured acceleration, and mental health could relate to the nature of device-measured movement behaviours capturing more activity than that from self-report measures, which might disproportionately focus respondents’ perceptions and recall on discrete episodes of physical activity, such as exercise and sport, at the expense of incidental activity. Without domain-specific physical activity information, it is possible that incidental activity across the intensity spectrum could attenuate potential beneficial effects of higher intensity physical activity (i.e., MPA and VPA) accrued during structured and unstructured exercise and sports [75]. Moreover, it not uncommon for accelerometers to be removed during exercise and sports for safety reasons [76], which would further mask potential beneficial influences of MPA and/or VPA on mental health.

### Aim 3

The optimal daily compositions of sleep, ST, LPA, MPA, and VPA for overall mental health and externalising problems had subtle differences from each other and were characterised by longer sleep, more LPA, and VPA, and less ST and MPA, relative to the sample mean composition. The only other published study of optimal daily time-use compositions and mental health in youth reported associations with SDQ emotional problems and depressive symptoms [18], which were not included in our Aim 3 analysis because internalising problems were not significantly associated with the mean sample composition in Aim 2. Notwithstanding this and in the absence of other comparable data, we highlight the consistent important contribution of sleep, irrespective of mental health outcome under consideration. In Dumuid et al.’s Australian sample, 582 min•day^− 1^ (9.7 h•day^− 1^) was optimised for emotional problems and depressive symptoms [18], compared to 600 min•day^− 1^ (10 h•day^− 1^; overall mental health) and 564 min•day^− 1^ (9.4 h•day^− 1^; externalising problems) in our analyses. Displacing ST with LPA seemed to be important for overall mental health and externalising problems in our analyses, whereas Dumuid and colleagues found that relatively less LPA and more MVPA were optimal for emotional problems and depressive symptoms [18]. Hypothetical optimal time-use compositions for mental health outcomes have much merit and through data visualisations such as those presented here and elsewhere [18, 77], can be extremely useful in translating key messages to research users (e.g., policy makers) and others with an influence over children’s movement behaviours, such as parents and teachers [78]. The optimal compositions though, are sample-specific and may vary according to methods used to measure time-use exposures and mental health outcomes. Thus, caution is urged when comparing them across between studies. Studies involving larger nationally representative samples are required to further investigate the optimal time-use compositions for indicators of mental health.

This novel study has several strengths. We used unfiltered raw acceleration data from wrist-accelerometers to assess 24-hour movement behaviour time-use estimates, sleep quality, 24-hour rest-activity rhythm, and directly measured acceleration metrics. We employed stringent wear time criteria which ensured all participants in the analytical sample wore the device for 24 h•day^− 1^ for a minimum of four days, including at least one weekend day. On average the participants had 6.5 valid days wear, indicating strong compliance to the protocol and a high degree of reliability in the resultant data. Further, mental health outcomes were assessed using a validated and well-established self-report tool and we applied the innovative ‘Goldilocks’ compositional analysis approach to generate optimal time-use compositions that were specific to different mental health outcomes. The study also had limitations which should be considered when interpreting the findings. The sample size was not large or representative beyond the region where the research took place. To a degree the sample size was reflective of the strict accelerometer inclusion criteria which increased data attrition, but arguably enhanced the reliability of the movement behaviour data. There was a risk of sampling bias because of observed differences in included and excluded participants who were older and from lower SES families. For these reasons generalising the findings beyond the analytical sample should be done with caution. Moreover, the cross-sectional design precludes any claims of causal inference and directionality between the 24-hour movement behaviour characteristics and mental health outcomes. Lastly, although the analyses were adjusted for variables that are known to influence the exposures and outcomes, we cannot rule out other sources of unmeasured residual confounding.

## Conclusions

24-hour movement behaviour characteristics among children and adolescents differed most in relation to time-use estimates, directly measured acceleration, and some 24-hour rest-activity rhythm metrics. Overall mental health and externalising problems but not internalising problems were associated with various 24-hour movement behaviour characteristics reflecting time-use estimates, sleep quality, and 24-hour rest-activity rhythms. Optimal time-use compositions for overall mental health and externalising problems were characterised by high sleep and LPA, and low ST, MPA, and VPA relative to the mean sample composition. Developing population-level optimal time-use compositions for a range of mental (and physical) health outcomes may further our understanding of how to target movement behaviours through interventions. Extracting and examining multiple movement behaviour characteristics from 24-hour accelerometer data can provide a more rounded picture of the interplay between different elements of movement behaviours and their relationships with health and wellbeing than single characteristics alone, such as time-use estimates. Applying multiple movement behaviour characteristics to the translation of research findings in this way may enhance the ability of research users to use research data to inform mental health-promotion programmes and services.

## Electronic supplementary material

Below is the link to the electronic supplementary material.

Additional file 1 Table S1. Summary of aim 2 linear mixed model results.

Additional file 2 Table S2. Geometric means of mean time-use composition; Table S3. Compositional variation matrix time-use estimates.

Additional file 3 STROBE checklist.

## Data Availability

The dataset analysed during the current study is available in the Open Science Framework repository from https://osf.io/2az8b.

## List of Abbreviations

AvAcc: Average acceleration
BMI: Body mass index
BMIz: Body mass index z-score
EIMD: English Indices of Multiple Deprivation
ENMO: Euclidean Norm Minus One
IS: Inter-daily stability
IV: Intra-daily variability
L5: Least active 5 h
LPA: Light physical activity
M10: Most active 10 h
MPA: Moderate physical activity
MVPA: Moderate-to-vigorous physical activity
SDQ: Strengths and Difficulties Questionnaire
ST: Sedentary time
VIF: Variance inflation factor
VPA: Vigorous physical activity

## References

[1] Tremblay MS, Carson V, Chaput J-P, Connor Gorber S, Dinh T, Duggan M, et al. Canadian 24-Hour Movement Guidelines for Children and Youth: an integration of physical activity, sedentary behaviour, and sleep. Appl Physiol Nutr Metab. 2016;41(6):311–S27. (Suppl. 3)). 10.1139/apnm-2016-0203 27306430

[2] New Zealand Ministry of Health. New Zealand Physical Activity Guidelines 2017. https://www.health.govt.nz/our-work/preventative-health-wellness/physical-activity#kids. Accessed 1 November 2022.

[3] Australian Government Department of Health. Australian 24-Hour Movement Guidelines for Children and Young People (5–17 years) - An Integration of Physical Activity, Sedentary Behaviour and Sleep 2019. https://www1.health.gov.au/internet/main/publishing.nsf/Content/health-24-hours-phys-act-guidelines. Accessed 1 November 2022.

[4] Loo BKG, Okely AD, Pulungan A, Jalaludin MY. Asia-Pacific Consensus Statement on integrated 24-hour activity guidelines for children and adolescents. Br J Sports Med. 2022;56(10):539–45. 34750119 10.1136/bjsports-2021-104527

[5] Tapia-Serrano MA, Sevil-Serrano J, Sánchez-Miguel PA, López-Gil JF, Tremblay MS, García-Hermoso A. Prevalence of meeting 24-Hour Movement Guidelines from pre-school to adolescence: a systematic review and meta-analysis including 387,437 participants and 23 countries. J Sport Health Sci. 2022. 10.1016/j.jshs.2022.01.005. 35066216 10.1016/j.jshs.2022.01.005 PMC9338333

[6] Saunders TJ, Gray CE, Poitras VJ, Chaput JP, Janssen I, Katzmarzyk PT, et al. Combinations of physical activity, sedentary behaviour and sleep: relationships with health indicators in school-aged children and youth. Appl Physiol Nutr Metab. 2016;41(6 Suppl 3):283–93. 10.1139/apnm-2015-0626 27306434

[7] Wilhite K, Booker B, Huang B-H, Antczak D, Corbett L, Parker P, et al. Combinations of physical activity, sedentary behavior, and sleep and their associations with physical, psychological, and educational outcomes in children and adolescents: a systematic review. Am J Epidemiol. 2022. 10.1093/aje/kwac212. 10.1093/aje/kwac212 PMC10089066 36516992

[8] Thivel D, Tremblay MS, Katzmarzyk PT, Fogelholm M, Hu G, Maher C, et al. Associations between meeting combinations of 24-hour movement recommendations and dietary patterns of children: a 12-country study. Prev Med. 2019;118:159–65. 30393016 10.1016/j.ypmed.2018.10.025

[9] Carson V, Chaput JP, Janssen I, Tremblay MS. Health associations with meeting new 24-hour movement guidelines for canadian children and youth. Prev Med. 2017;95:7–13. 27923668 10.1016/j.ypmed.2016.12.005

[10] Katzmarzyk PT, Staiano AE. Relationship between meeting 24-Hour Movement Guidelines and Cardiometabolic Risk factors in children. J Phys Act Health. 2017;14(10):779–84. 28556685 10.1123/jpah.2017-0090 PMC5607096

[11] Sampasa-Kanyinga H, Colman I, Goldfield GS, Janssen I, Wang J, Podinic I, et al. Combinations of physical activity, sedentary time, and sleep duration and their associations with depressive symptoms and other mental health problems in children and adolescents: a systematic review. Int J Behav Nutr Phys Activity. 2020;17(1):72. 10.1186/s12966-020-00976-x PMC7273653 32503638

[12] Migueles JH, Aadland E, Andersen LB, Brønd JC, Chastin SF, Hansen BH et al. GRANADA consensus on analytical approaches to assess associations with accelerometer-determined physical behaviours (physical activity, sedentary behaviour and sleep) in epidemiological studies.Br J Sports Med. 2021; bjsports-2020-103604. 10.1136/bjsports-2020-103604 PMC8938657 33846158

[13] Dumuid D, Pedišić Ž, Palarea-Albaladejo J, Martín-Fernández JA, Hron K, Olds T. Compositional data analysis in time-use epidemiology: what, why, how. Int J Env Res Public Health. 2020;17(7):2220. 32224966 10.3390/ijerph17072220 PMC7177981

[14] Fairclough SJ, Dumuid D, Taylor S, Curry W, McGrane B, Stratton G, et al. Fitness, fatness and the reallocation of time between children’s daily movement behaviours: an analysis of compositional data. Int J Behav Nutr Phys Activity. 2017;14(1):64. 10.1186/s12966-017-0521-z PMC5424384 28486972

[15] Dumuid D, Simm P, Wake M, Burgner D, Juonala M, Wu F, et al. The “Goldilocks Day” for children’s skeletal health: compositional data analysis of 24-hour activity behaviors. J Bone Mineral Research. 2020. 10.1002/jbmr.4143. 10.1002/jbmr.4143 32730680

[16] Fairclough SJ, Tyler R, Dainty JR, Dumuid D, Richardson C, Shepstone L et al. Cross-sectional associations between 24-hour activity behaviours and mental health indicators in children and adolescents: A compositional data analysis. J Sports Sci. 2021:1–13. 10.1080/02640414.2021.1890351 33615990

[17] Chong KH, Parrish AM, Cliff DP, Dumuid D, Okely AD. Cross-Sectional and longitudinal associations between 24-hour movement behaviours, recreational screen use and psychosocial health outcomes in children: a compositional data analysis approach. Int J Environ Res Public Health. 2021;18(11). 10.3390/ijerph18115995 PMC8199728 34204928

[18] Dumuid D, Olds T, Lange K, Edwards B, Lycett K, Burgner DP, et al. Goldilocks Days: optimising children’s time use for health and well-being. J Epidemiol Comm Health. 2022;76(3):301–8. 10.1136/jech-2021-216686 34385290

[19] van Hees VT, Sabia S, Jones SE, Wood AR, Anderson KN, Kivimäki M, et al. Estimating sleep parameters using an accelerometer without sleep diary. Sci Rep. 2018;8(1):12975. 30154500 10.1038/s41598-018-31266-z PMC6113241

[20] Neikrug AB, Chen IY, Palmer JR, McCurry SM, Von Korff M, Perlis M, et al. Characterizing behavioral activity rhythms in older adults using actigraphy. Sensors. 2020;20(2):549. 31963889 10.3390/s20020549 PMC7014517

[21] Rowlands AV, Edwardson CL, Davies MJ, Khunti K, Harrington DM, Yates T. Beyond cut-points: accelerometer metrics that capture the physical activity profile. Med Sci Sports Exerc. 2018;50(6):1323–32. 29360664 10.1249/MSS.0000000000001561

[22] Lovato N, Gradisar M. A meta-analysis and model of the relationship between sleep and depression in adolescents: recommendations for future research and clinical practice. Sleep Med Rev. 2014;18(6):521–9. 24857255 10.1016/j.smrv.2014.03.006

[23] Cortese S, Faraone SV, Konofal E, Lecendreux M. Sleep in children with attention-deficit/hyperactivity disorder: meta-analysis of subjective and objective studies. J Am Acad Child Adol Psychiatry. 2009;48(9):894–908. 10.1097/CHI.0b013e3181ac09c9 19625983

[24] Blok E, Koopman-Verhoeff ME, Dickstein DP, Saletin J, Luik AI, Rijlaarsdam J, et al. Sleep and mental health in childhood: a multi-method study in the general pediatric population. Child Adol Psychiatry Mental Health. 2022;16(1):11. 10.1186/s13034-022-00447-0 PMC8851725 35177100

[25] Crowley SJ, Wolfson AR, Tarokh L, Carskadon MA. An update on adolescent sleep: new evidence informing the perfect storm model. J Adol. 2018;67(1):55–65. 10.1016/j.adolescence.2018.06.001 PMC6054480 29908393

[26] Thompson D, Batterham AM. Towards integrated physical activity profiling. PLoS ONE. 2013;8(2):e56427. 23437131 10.1371/journal.pone.0056427 PMC3577906

[27] Rowlands AV, Edwardson CL, Dawkins NP, Maylor BD, Metcalf KM, Janz KF. Physical activity for Bone Health: how much and/or how hard? Med Sci Sports Exerc. 2020;52(11):2331–41. 32453172 10.1249/MSS.0000000000002380

[28] Fairclough SJ, Taylor S, Rowlands AV, Boddy LM, Noonan RJ. Average acceleration and intensity gradient of primary school children and associations with indicators of health and well-being. J Sports Sci. 2019;37(18):2159–67. 31156048 10.1080/02640414.2019.1624313

[29] Pedisic Z, Dumuid D, Olds T. Integrating sleep, sedentary behaviour, and physical activity research in the emerging field of time-use epidemiology: definitions, concepts, statistical methods, theoretical framework, and future directions. Kinesiology. 2017;49(2):252–69.

[30] Lohman TG, Roche AFM, Martorell R. Anthropometric standardization reference manual. Illinois: Champaign, IL: Human Kinetics Books; 1991.

[31] Cole T, Freeman J, Preece M. Body mass index reference curves for the UK, 1990. Arch Dis Child. 1995;73(1):25–9. 7639544 10.1136/adc.73.1.25 PMC1511150

[32] Cole T, Bellizzi M, Flegal K, Dietz W. Establishing a standard definition for child overweight and obesity worldwide: international survey. Br Med J. 2000;320:1240–3. 10797032 10.1136/bmj.320.7244.1240 PMC27365

[33] English Indices of Deprivation. 2019. 2019. http://imd-by-postcode.opendatacommunities.org/imd/2019. Accessed 9 June 2022.

[34] Goodman R. Psychometric properties of the Strengths and Difficulties Questionnaire. J Am Acad Child Adolesc Psychiatry. 2001;40(1):1337–45. 11699809 10.1097/00004583-200111000-00015

[35] Goodman A, Lamping DL, Ploubidis GB. When to use broader internalising and externalising subscales instead of the hypothesised five subscales on the Strengths and Difficulties Questionnaire (SDQ): data from british parents, teachers and children. J Abnorm Child Psychol. 2010;38(8):1179–91. 20623175 10.1007/s10802-010-9434-x

[36] Youth in Mind. Scoring the Strengths and Difficulties Questionnaire for age 4–17 or 18 + 2016. https://www.sdqinfo.org/py/sdqinfo/c0.py. Accessed 9 June 2022.

[37] Migueles JH, Rowlands AV, Huber F, Sabia S, Van Hees VT, GGIR. A research community–driven open source r package for generating physical activity and sleep outcomes from multi-day raw accelerometer data. J Meas Phys Behav. 2019;2:188–96.

[38] van Hees VT, Fang Z, Langford J, Assah F, Mohammad A, da Silva IC, et al. Autocalibration of accelerometer data for free-living physical activity assessment using local gravity and temperature: an evaluation on four continents. J Appl Physiol. 2014;117(7):738–44. 25103964 10.1152/japplphysiol.00421.2014 PMC4187052

[39] van Hees VT, Gorzelniak L, León EC, Eder M, Pias Mo, Taherian S, et al. Separating movement and gravity components in an acceleration signal and implications for the assessment of human daily physical activity. PLoS ONE. 2013;8(4):e61691. 23626718 10.1371/journal.pone.0061691 PMC3634007

[40] Hurter L, Fairclough SJ, Knowles ZR, Porcellato LA, Cooper-Ryan AM, Boddy LM. Establishing raw acceleration thresholds to classify sedentary and stationary behaviour in children. Children. 2018;5(12). 10.3390/children5120172 PMC6306859 30572683

[41] Hildebrand M, Van Hees VT, Hansen BH, Ekelund U. Age-group comparibility of raw accelerometer output from wrist- and hip-worn monitors. Med Sci Sports Exerc. 2014;46(9):1816–24. 24887173 10.1249/MSS.0000000000000289

[42] Di J, Spira A, Bai J, Urbanek J, Leroux A, Wu M, et al. Joint and individual representation of domains of physical activity, sleep, and circadian rhythmicity. Stat Biosci. 2019;11(2):371–402. 32440309 10.1007/s12561-019-09236-4 PMC7241438

[43] Neikrug AB, Donaldson G, Iacob E, Williams SL, Hamilton CA, Okifuji A. Activity rhythms and clinical correlates in fibromyalgia. Pain. 2017;158(8):1417–29. 28328573 10.1097/j.pain.0000000000000906 PMC5515675

[44] n den Boogaart KG, Tolosana-Delgado R. Compositions’: a unified R package to analyze compositional data. Comput Geosci. 2008;34(4):320–38.

[45] Dumuid D, Stanford TE, Martin-Fernandez JA, Pedisic Z, Maher CA, Lewis LK et al. Compositional data analysis for physical activity, sedentary time and sleep research. Stat Methods Med Res. 2017:962280217710835. 10.1177/0962280217710835 28555522

[46] Bang F, Roberts KC, Chaput JP, Goldfield GS, Prince SA. Physical activity, screen time and sleep duration: combined associations with psychosocial health among canadian children and youth. Health Rep. 2020;31:9–16. 32644766 10.25318/82-003-x202000500002-eng

[47] Kuznetsova A, Brockhoff PB, Christensen RHB. lmerTest Package: tests in Linear mixed Effects Models. J Stat Softw. 2017;82(13):26.

[48] Fox J, Weisberg S, An R. Companion to Applied Regression. Thousand Oaks, CA: Sage; 2019.

[49] Ludecke D, Ben-Shachar MS, Patil I, Waggoner P, Makowski D. Performance: an R package for assessment, comparison and testing of statistical models. J Open Sci Software. 2021;60(60):3139.

[50] Youth in Mind. Scoring the SDQ 2016. https://www.sdqinfo.org/py/sdqinfo/c0.py. Accessed 9 June 2022.

[51] Rowlands AV, Dawkins NP, Maylor B, Edwardson CL, Fairclough SJ, Davies MJ, et al. Enhancing the value of accelerometer-assessed physical activity: meaningful visual comparisons of data-driven translational accelerometer metrics. Sports Med Open. 2019;5(1):47. 31808014 10.1186/s40798-019-0225-9 PMC6895365

[52] Cooper AR, Goodman A, Page AS, Sherar LB, Esliger DW, van Sluijs EM, et al. Objectively measured physical activity and sedentary time in youth: the international children’s accelerometry database (ICAD). Int J Behav Nutr Phys Act. 2015;12:113. 26377803 10.1186/s12966-015-0274-5 PMC4574095

[53] Rowlands AV, Fairclough SJ, Yates TOM, Edwardson C, Davies M, Munir F, et al. Activity intensity, volume, and norms: utility and interpretation of accelerometer metrics. Med Sci Sports Exerc. 2019;51(11):2410–22. 31318713 10.1249/MSS.0000000000002047

[54] Mitchell JA, Quante M, Godbole S, James P, Hipp JA, Marinac CR, et al. Variation in actigraphy-estimated rest-activity patterns by demographic factors. Chronobiol Int. 2017;34(8):1042–56. 28650674 10.1080/07420528.2017.1337032 PMC6101244

[55] Sherar LB, Esliger DW, Baxter-Jones AD, Tremblay MS. Age and gender differences in youth physical activity: does physical maturity matter? Med Sci Sports Exerc. 2007;39(5):830–5. 17468582 10.1249/mss.0b013e3180335c3c

[56] Hilland TA, Costigan SA. Children’s and adolescents’ interpersonal-level correlates of physical activity behavior. In: Brusseau TA, Fairclough SJ, Lubans DR, editors. The Routledge Handbook of Youth Physical Activity. London: Routledge; 2020. pp. 191–212.

[57] Waldhauser KJ, Ruissen GR, Beauchamp MR. Psychological factors associated wkith physical activity in youth. In: Brusseau TA, Fairclough SJ, Lubans DR, editors. The Routledge Handbook of Youth Physical Activity. London: Routledge; 2020. pp. 173–90.

[58] Dumuid D, Stanford TE, Pedišić Ž, Maher C, Lewis LK, Martín-Fernández J-A, et al. Adiposity and the isotemporal substitution of physical activity, sedentary time and sleep among school-aged children: a compositional data analysis approach. BMC Public Health. 2018;18(1):311. 29499689 10.1186/s12889-018-5207-1 PMC5834855

[59] Galland BC, Short MA, Terrill P, Rigney G, Haszard JJ, Coussens S et al. Establishing normal values for pediatric nighttime sleep measured by actigraphy: a systematic review and meta-analysis. Sleep. 2018;41(4). 10.1093/sleep/zsy017 29590464

[60] Elkhatib Smidt SD, Hitt T, Zemel BS, Mitchell JA. Sex differences in childhood sleep and health implications. Ann Hum Biol. 2021;48(6):474–84. 35105205 10.1080/03014460.2021.1998624 PMC9254351

[61] Ohayon M, Wickwire EM, Hirshkowitz M, Albert SM, Avidan A, Daly FJ, et al. National Sleep Foundation’s sleep quality recommendations: first report. Sleep Health. 2017;3(1):6–19. 28346153 10.1016/j.sleh.2016.11.006

[62] Paruthi S, Brooks LJ, D’Ambrosio C, Hall WA, Kotagal S, Lloyd RM, et al. Recommended amount of sleep for pediatric populations: a consensus statement of the American Academy of Sleep Medicine. J Clin Sleep Med. 2016;12(6):785–6. 27250809 10.5664/jcsm.5866 PMC4877308

[63] Barth Vedøy I, Skulberg KR, Anderssen SA, Fagerland MW, Tjomsland HE, Thurston M. The longitudinal association between objectively measured physical activity and mental health among norwegian adolescents. Int J Behav Nutr Phys Activity. 2021;18(1):149. 10.1186/s12966-021-01211-x PMC8594230 34784906

[64] Inchley J, Currie D, Budisavljevic S, Torsheim T, Jåstad A. School-aged Children (HBSC) survey in Europe and Canada. International report. Volume 1. Key Findings. Copenhagen: WHO Regional Office for Europe; 2020. A C, et al. Spotlight on adolescent health and well-being. Findings from the 2017/2018 Health Behaviour in.

[65] Merikangas KR, Nakamura EF, Kessler RC. Epidemiology of mental disorders in children and adolescents. Dialogues Clin Neurosci. 2009;11(1):7–20. 19432384 10.31887/DCNS.2009.11.1/krmerikangas PMC2807642

[66] Sport England. Active lives children and Young People Survey. Academic Year 2020-21. London: Sport England; 2021.

[67] Ravens-Sieberer U, Kaman A, Erhart M, Devine J, Schlack R, Otto C. Impact of the COVID-19 pandemic on quality of life and mental health in children and adolescents in Germany. Eur Child Adol Psychiatry. 2021. 10.1007/s00787-021-01726-5 PMC7829493 33492480

[68] Gilchrist JD, Battista K, Patte KA, Faulkner G, Carson V, Leatherdale ST. Effects of reallocating physical activity, sedentary behaviors, and sleep on mental health in adolescents. Mental Health Phys Activity. 2021;20:100380.

[69] Asare M. Sedentary behaviour and mental health in children and adolescents: a meta-analysis. J Child Adol Behav. 2015. 10.4172/2375-4494.1000259.

[70] Youth in Mind. SDQ: Normative school-age SDQ data from Britain 2016. https://www.sdqinfo.org/norms/UKSchoolNorm.html. Accessed 9 June 2022.

[71] Qiu J, Morales-Muñoz I. Associations between sleep and mental health in adolescents: results from the UK Millennium Cohort Study. Int J Env Res Public Health. 2022;19(3):1868. 35162890 10.3390/ijerph19031868 PMC8835146

[72] Rabinowitz JA, An Y, He L, Alfini AJ, Zipunnikov V, Wu MN, et al. Associations of circadian rest/activity rhythms with cognition in middle-aged and older adults: demographic and genetic interactions. Front Neurosci. 2022. 10.3389/fnins.2022.952204. 36312032 10.3389/fnins.2022.952204 PMC9597505

[73] Kelly Y, Kelly J, Sacker A. Changes in bedtime schedules and behavioral difficulties in 7 year old children. Pediatrics. 2013;132(5):e1184–e93. 24127471 10.1542/peds.2013-1906

[74] Kelly Y, Kelly J, Sacker A. Time for bed: associations with cognitive performance in 7-year-old children: a longitudinal population-based study. J Epidemiol Comm Health. 2013;67(11):926–31. 10.1136/jech-2012-202024 PMC3812865 23835763

[75] Colley RC, Butler G, Garriguet D, Prince SA, Roberts KC. Comparison of self-reported and accelerometer-measured physical activity among canadian youth. Health Rep. 2019;30(7):3–12. 31314124 10.25318/82-003-x201900700001-eng

[76] Koorts H, Timperio A, Arundell L, Parker K, Abbott G, Salmon J. Is sport enough? Contribution of sport to overall moderate- to vigorous-intensity physical activity among adolescents. J Sci Med Sport. 2019;22(10):1119–24. 31277920 10.1016/j.jsams.2019.06.009 PMC8863377

[77] Dumuid D, Olds T, Wake M, Lund Rasmussen C, Pedišić Ž, Hughes JH, et al. Your best day: an interactive app to translate how time reallocations within a 24-hour day are associated with health measures. PLoS ONE. 2022;17(9):e0272343. 36070284 10.1371/journal.pone.0272343 PMC9451088

[78] Stanley R, Jones R, Swann C, Christian H, Sherring J, Shilton T, et al. Exploring stakeholders’ perceptions of the acceptability, usability, and dissemination of the australian 24-Hour Movement Guidelines for the early years. J Phys Activity Health. 2020;17(1):120–5. 10.1123/jpah.2019-0069 31357261

