# Supplementary material for: Characteristics of 24-hour movement behaviours and their associations with mental health in children and adolescents
Source: J Act Sedentary Sleep Behav. 2023 Jun 2;2:11. doi: 10.1186/s44167-023-00021-9 (PMC10234795; doi:10.1186/s44167-023-00021-9)
Supplement: Supplementary file 1 — Additional file 1 Table S1. Summary of aim 2 linear mixed model results. [file 44167_2023_21_MOESM1_ESM.docx]

Additional file 1

Table S1. Summary of aim 2 linear mixed model results

|  | Overall mental health |  |  | Externalising problems |  |  | Internalising problems |  |  |
| --- | --- | --- | --- | --- | --- | --- | --- | --- | --- |
| Characteristic | β | SE | p | β | SE | p | β | SE | p |
| Time-use |  |  |  |  |  |  |  |  |  |
| Sleep | -6.0 | 2.5 | .01 | -3.9 | 1.4 | .01 |  |  |  |
| ST | 4.7 | 1.7 | .01 | 2.6 | 1.0 | .01 |  |  |  |
| LPA | 0.1 | 2.3 | .70 | -0.03 | 1.4 | .98 |  |  |  |
| MPA | 1.4 | 1.8 | .44 | 1.8 | 1.0 | .09 |  |  |  |
| VPA | -0.8 | 0.8 | .27 | -0.4 | 0.4 | 0.3 |  |  |  |
| Sleep quality |  |  |  |  |  |  |  |  |  |
| Sleep efficiency | -18.0 | 8.6 | .04 | -12.8 | 5.0 | .01 | -5.3 | 5.2 | .31 |
| Night awakenings | -0.2 | 0.1 | .06 | -0.1 | 0.06 | .05 | -0.1 | 0.1 | .23 |
| Sleep onset time | 0.2 | 0.3 | .40 | .08 | 0.2 | .63 | 0.2 | 0.2 | .28 |
| 24-hour rest-activity rhythms |  |  |  |  |  |  |  |  |  |
| Mesor | -0.5 | 1.5 | .78 | 0.03 | 0.9 | .98 | -0.5 | 0.9 | .58 |
| Amplitude | 1.0 | 0.5 | .03 | 0.7 | 0.3 | .02 | 0.4 | 0.3 | .18 |
| Acrophase | -0.2 | 0.6 | .70 | -0.1 | 0.3 | .82 | -0.1 | 0.3 | .75 |
| IS | -9.9 | 4.3 | .02 | -6.7 | 2.5 | .009 | -3.5 | 2.6 | .18 |
| IV | 3.3 | 2.6 | .20 | -2.3 | 1.5 | .13 | -1.2 | 1.5 | .44 |
| L5 | -0.1 | 0.3 | .41 | -0.1 | 0.1 | .21 | -0.01 | 0.1 | .92 |
| M10 | -.01 | 0.2 | .52 | 0.01 | 0.01 | .59 | -0.02 | 0.01 | .10 |
| Directly measured acceleration |  |  |  |  |  |  |  |  |  |
| Average acceleration | -0.02 | 0.03 | .05 | 0.01 | 0.02 | .81 | -0.03 | 0.02 | .14 |
| Intensity gradient | -1.59 | 2.64 | .54 | -0.1 | 1.6 | .95 | -1.7 | 1.6 | .28 |

Note. The mean time-use composition was not significantly associated with internalising problems, therefore follow-up analyses were not performed. ST=sedentary time; LPA=light physical activity; MPA=moderate physical activity; VPA=vigorous physical activity; IS=inter-daily stability; IV=intra-daily variability; L5=activity level during the least active 5 hours; M10=activity level during the most active 10 hours.
