# Supplementary material for: Characteristics of 24-hour movement behaviours and their associations with mental health in children and adolescents
Source: J Act Sedentary Sleep Behav. 2023 Jun 2;2:11. doi: 10.1186/s44167-023-00021-9 (PMC10234795; doi:10.1186/s44167-023-00021-9)
Supplement: Supplementary file 2 — Additional file 2 Table S2. Geometric means of mean time-use composition; Table S3. Compositional variation matrix time-use estimates. [file 44167_2023_21_MOESM2_ESM.docx]

Additional file 2

Table S2. Geometric means of mean time-use composition

|  | Sleep | ST | LPA | MPA | VPA |
| --- | --- | --- | --- | --- | --- |
| Percentage of 24-hours | 34.3 | 40.9 | 20.7 | 3.7 | 0.5 |
| min⋅day^-1^ | 493.8 | 588.5 | 298.2 | 52.6 | 6.8 |
| hour⋅day^-1^ | 8.2 | 9.8 | 5.0 | 0.9 | 0.1 |

Note. ST=sedentary time; LPA=light physical activity; MPA=moderate physical activity; VPA=vigorous physical activity

Table S3. Compositional variation matrix time-use estimates

|  | Sleep | ST | LPA | MPA | VPA |
| --- | --- | --- | --- | --- | --- |
| Sleep | 0.00 | 0.08 | 0.07 | 0.21 | 0.70 |
| ST | 0.08 | 0.00 | 0.18 | 0.38 | 0.98 |
| LPA | 0.07 | 0.18 | 0.00 | 0.10 | 0.57 |
| MPA | 0.21 | 0.38 | 0.10 | 0.00 | 0.27 |
| VPA | 0.70 | 0.98 | 0.57 | 0.27 | 0.00 |

Note. ST=sedentary time; LPA=light physical activity; MPA=moderate physical activity; VPA=vigorous physical activity
