# Supplementary material for: Characteristics of 24-hour movement behaviours and their associations with mental health in children and adolescents
Source: J Act Sedentary Sleep Behav. 2023 Jun 2;2:11. doi: 10.1186/s44167-023-00021-9 (PMC10234795; doi:10.1186/s44167-023-00021-9)
Supplement: Supplementary file 3 — Additional file 3 STROBE checklist. [file 44167_2023_21_MOESM3_ESM.doc]

STROBE Statement—Checklist of items that should be included in reports of ***cross-sectional studies***

|  | Item No | Recommendation |
| --- | --- | --- |
| **Title and abstract** | 1 | (*a*) See title and abstract |
|  |  | (*b*) See abstract |
| Introduction | | |
| Background/rationale | 2 | See lines 58-127 |
| Objectives | 3 | See the lines 127-133 of the Background section |
| Methods | | |
| Study design | 4 | Cross-sectional design; See lines 138-145 of the Methods section |
| Setting | 5 | See lines 138-143 of the Methods section |
| Participants | 6 | See lines 138-143 of the Methods section and lines 286-294 of the Results section |
| Variables | 7 | See lines 149-237 of the Methods section |
| Data sources/ measurement | 8* | See lines 149-237 of the Methods section |
| Bias | 9 | Selection bias: The target group was primary and secondary school children and adolescents from northwest England. The participants were recruited from state schools in northwest England situated in a variety of urban and rural and socioeconomic locations. Participants were recruited by Year group and classes, and classes typically consisted of mixed ability students. Recruitment rates were high (typically ≥80%). For these reasons we are confident that the sample was representative of the target group and risk of selection bias was low.  Information bias: Accelerometer assessment protocols were standardised using accelerometers worn on the non-dominant wrist. Equivalence in raw acceleration output has been demonstrated between the two types of accelerometer used in the contributing studies. All raw accelerometer data were processed following a calibration procedure using the identical parameters and arguments in the same version of the GGIR R application. Accelerometer wear compliance was high based on stringent criterion of 24 hours/day wear as a valid day, and at least 3 valid weekdays and 1 valid weekend day of wear. |
| Study size | 10 | Study size was arrived at by initial consideration of all eligible data from the contributing studies (n=490), the removal of missing data (n=23), and accelerometer data that did not meet the wear time criteria (n=166) |
| Quantitative variables | 11 | See lines 149-237 of the Methods section |
| Statistical methods | 12 | (*a*) See Methods section ‘Data analysis’ |
|  |  | (*b*) See Methods section lines 242-282 |
|  |  | (*c*) Missing data were removed on a case-wise basis |
|  |  | (*d*) n/a |
|  |  | (*e*) n/a |
| Results | | |
| Participants | 13* | (a) See Results section lines 286-303 |
|  |  | (b) See Methods section lines 286-290 |
|  |  | (c) Not necessary as participant inclusion and exclusion were straightforward |
| Descriptive data | 14* | (a) See Table 2 |
|  |  | (b) The analytical sample had no missing data |
| Outcome data | 15* | See Table 3 |
| Main results | 16 | (*a*) See Tables 2 and 3, Figures 2 and 3, and Additional file 2 |
|  |  | (*b*) n/a |
|  |  | (*c*) n/a |
| Other analyses | 17 | n/a |
| Discussion | | |
| Key results | 18 | See Discussion section, paragraph 1 |
| Limitations | 19 | See Discussion section, lines 556-567 |
| Interpretation | 20 | See Conclusions section |
| Generalisability | 21 | See Discussion section, lines 557-563 |
| Other information | | |
| Funding | 22 | See Declarations section |

*Give information separately for exposed and unexposed groups.

**Note:** An Explanation and Elaboration article discusses each checklist item and gives methodological background and published examples of transparent reporting. The STROBE checklist is best used in conjunction with this article (freely available on the Web sites of PLoS Medicine at http://www.plosmedicine.org/, Annals of Internal Medicine at http://www.annals.org/, and Epidemiology at http://www.epidem.com/). Information on the STROBE Initiative is available at www.strobe-statement.org.
